# Evaluation and Comparison of Three Types of Spray Dried Coprocessed Excipient Avicel® for Direct Compression

**DOI:** 10.1155/2018/2739428

**Published:** 2018-04-19

**Authors:** Pavlína Vodáčková, Barbora Vraníková, Petra Svačinová, Aleš Franc, Jan Elbl, Jan Muselík, Roman Kubalák, Tomaš Solný

**Affiliations:** ^1^Department of Pharmaceutics, Faculty of Pharmacy, University of Veterinary and Pharmaceutical Sciences Brno, Palackého Tr. 1946/1, 612 42 Brno, Czech Republic; ^2^Department of Pharmaceutical Technology, Charles University, Faculty of Pharmacy in Hradec Kralove, Akademika Heyrovskeho 1203, 500 05 Hradec Kralove, Czech Republic; ^3^Materials Research Centre, Brno University of Technology, Faculty of Chemistry, Purkynova 464/118, 612 00 Brno, Czech Republic

## Abstract

As coprocessed excipients (CPE) gain a lot of focus recently, this article compares three commercially available CPE of Avicel brand, namely, CE 15, DG, and HFE 102. Comparison is based on measured physical properties of coprocessed mixtures, respectively, flow properties, pycnometric density, mean particle size, specific surface area, moisture content, hygroscopicity, solubility, pH leaching, electrostatic charge, SEM images, and DSC. Tablets were made employing three pressure sets. Viscoelastic properties and ejection force were assessed during compression, as well as pycnometric density, mass uniformity, height, tensile strength, friability, disintegration, and wetting times. Avicel CE 15 is of mid-range flow properties, contains mid-size and nonspherical particles, and has high hygroscopicity, growing negative charge, best lubricity, lowest tensile strength, and mid-long disintegration times. Avicel DG possesses the worst flow properties, small asymmetrical particles, lowest hygroscopicity, stable charge, intermediate lubricity, and tensile strength and exhibits fast disintegration of tablets. Finally, Avicel HFE 102 has the best flow properties, large symmetrical particles, and middle hygroscopicity and its charge fluctuates throughout blending. It also exhibits inferior lubricity, the highest tensile strength, and slow disintegration of tablets. Generally, it is impossible to select the best CPE, as their different properties fit versatile needs of countless manufacturers and final products.

## 1. Introduction

Tablets are the most frequent dosage form due to their advantages, for example, availability, easy administration, good stability, and low price. The easiest technology of production is still direct compression (DC), but this method requires overcoming many complications. These mostly include a problematic content and mass uniformity and low mechanical resistance. “Coprocessed excipients” (CPE), containing commonly processed blends of fillers, binders, disintegrants, lubricants, and other excipients, are nowadays more and more used and may help to overcome some disadvantages of DC. Using physically and structurally uniform mixture (tableting blend) of usually porous particles with suitable properties and defined particle size distribution enhances flowability, dilution potential, content uniformity, and tabletability, while reducing lubricant sensitivity and enabling better processability of different active pharmaceutical ingredients (API) into dosage forms [1, 2]. Results are tablets with enhanced content and mass uniformity, hardness, disintegration, decreased friability, and improved bioavailability of admixed API [3–6].

CPE are produced by different technologies of which the spray drying (SpD) is the most widely used. SpD forms highly porous particles with good flowability and tabletability ensuring short time of disintegration in the physiological fluids [2, 7, 8]. Usually spherical shape improves the flow properties and provides better rearrangement of the particles in the die during tableting, resulting in better compaction characteristics. However, although the composition of these CPE may be virtually similar, the small changes in the component's characteristics can make them behave differently after tableting [9].

The aim of this work was a comparison of three commercially available binary mixtures of CPE produced by SpD technology. These are Avicel CE 15, Avicel DG, and Avicel HFE 102 (FMC Health Nutrition) with different content of microcrystalline cellulose (MCC) and 15% of guar gum, 25% of dicalcium phosphate (DCPA), and 10% of mannitol, respectively. Contemporary data information coming from a few scientific articles and producer do not provide whole-proof compatibility because of different used methodology [10–14]. Therefore, these individual CPE were now evaluated for flow properties including angle of slide, sieve analysis, specific surface area, moisture content, hygroscopicity, solubility, pH leaching, electrostatic charge, and compressibility without lubricant. Mass uniformity, pycnometric density, height, tensile strength, friability, disintegration, wetting rate, and water absorption ratio were evaluated for the compressed tablets. SEM was performed for both CPE excipients and tablets. Some of results have never been published before by any other authors.

The aim of this work was to evaluate the properties of individual CPE. Since the specific properties of these individual excipients can be selectively overlapped by the addition of another substance according to their nature, no model drugs have been added. Obtained data may help producers to choose suitable excipients for tablet formulation and to find out dependency between composition and tablet behavior in oral cavity and/or gastrointestinal tract.

## 2. Materials and Methods

### 2.1. Materials

Avicel CE 15, Avicel DG, and Avicel HFE 102 were received as a kind gift from FMC Health Nutrition, USA.

### 2.2. Methods

#### 2.2.1. Flow Properties

The flow through the orifice of the CPE was measured on a flowability tester (Ing. Jaromír Havelka, Czech Republic) according to Ph. Eur. with an orifice diameter of 15 mm. Measurements were performed in triplicate and the results are expressed as mean values ± standard deviation.

Angle of repose was determined by measuring the height of the cone of powder (100 g) and calculating the angle of repose, *α*, according to Ph. Eur. Measurements were performed in triplicate and the results are expressed as mean values ± standard deviation.

Angle of slide was tested with powder sample (10 g) which was placed on one end of a metal plate with a polished surface. This end was gradually raised until the plate with the horizontal surface formed an angle at which the sample was about to slide [15]. Measurements were performed in triplicate and the results are expressed as mean values ± standard deviation.

Bulk and tapped volumes were evaluated in a tapped density tester (SVM 102, Erweka GmbH, Germany) and subsequently used to calculate bulk and tapped densities, Hausner ratio (HR), and Carr compressibility index (CI) according to Ph. Eur.

#### 2.2.2. Pycnometric Density and Porosity of Powders

True density of the CPE was determined by the gas displacement technique using the helium pycnometer (Pycnomatic ATC, Ing. Prager Elektronik Handels GmbH, Austria), according to Ph. Eur. All density measurements were performed in triplicate and the results are expressed as mean values ± standard deviation. Porosity of CPE was calculated according to the following equation:

(1) $$\begin{array}{c} porosity=\frac{\rho_{bulk}-\rho_{pycnometric}}{\rho_{pycnometric}}\cdot 100. \end{array}$$

#### 2.2.3. Sieve Analysis

Particle size distribution was evaluated by a sieve analysis using a set of stainless steel sieves with apertures ranging from 0.025 to 0.800 mm placed on a vibratory sieve shaker (AS 200 basic, Retsch GmbH & Co. KG, Ingelheim, Germany). The percentage weight of tablets retained on each of the sieves was determined.

#### 2.2.4. Mean Particle Size

Particle size was determined by laser diffraction of dry samples (HELOS KR, SYMPATEC GmbH, Germany). *D*_10_, *D*_50_, and *D*_90_ are the diameters of sample at the 10th, 50th, and 90th percentiles of the cumulative percent undersize plot. Measurements were performed in duplicate and the results are expressed as mean values ± standard deviation.

#### 2.2.5. Specific Surface Area

The specific surface area was determined by nitrogen adsorption (MSP, Geotest Brno, Czech Republic). Samples were degassed at 200°C in vacuum for 24 h before the measurement. The specific surface area was obtained from the Brunauer-Emmett-Teller (BET) model [16] using 0.162 nm^2^ as the area occupied by one nitrogen molecule. Powdered titan oxide (SSA = 13.76 m^2^/g) was used as standard. Measurements were performed in triplicate and the results are expressed as mean values ± standard deviation.

#### 2.2.6. Moisture Content

The percentage of moisture content in the coprocessed excipients was assayed in a halogen moisture analyzer (Mettler Toledo, HX204, Switzerland) under the following conditions: standard drying program, drying temperature 105°C, and switch-off criterion 1 mg·50 s^−1^. Measurements were performed in duplicate and the results are expressed as mean values ± standard deviation.

#### 2.2.7. Hygroscopicity

The hygroscopicity was measured in constant climate chamber (Binder, KBF 240, Germany) under the following conditions: temperature of 40°C, humidity of 75% RV, and duration of 30 days. 3 grams of the samples were examined in 0.25, 0.5, 1, 3, 5, 8, 24, 72, 120, 168, and 720 hours in a halogen moisture analyzer (Mettler Toledo, HX204, Switzerland). Measurements were performed in duplicate and the results are expressed as mean values ± standard deviation.

#### 2.2.8. Solubility

The percentage of soluble fraction of CPE was determined. The first phase was to evaluate the insoluble fraction at physiological amount of saliva (15 mL) in mouth to simulate oral cavity. One gram of the sample was dried in hot air dryer (Horo, Type 38A, Germany) at 60°C for 4 hours. After drying it was weighed and dissolved in 15 mL of artificial saliva by stirring (600 rounds per minute) in mechanical stirrer (HEIDOLPH RZR 2021, Sigma Aldrich, USA) for 3 min. Then it was filtered through a filter paper dried in hot air dryer at 60°C for 4 hours. The filter paper with the captured sample was dried again in hot air dryer at 60°C for 4 hours. The percentage of undissolved fraction was calculated from the weight difference of filter paper. Measurements were performed in duplicate and the results are expressed as mean values ± standard deviation.

The second phase was to determine the total soluble fraction. One gram of the sample was dried in hot air oven (60°C, 4 h) and dissolved in 900 mL of artificial saliva by stirring (600 rounds per minute) in mechanical stirrer for 24 hours. Artificial saliva was prepared according to the formula as observed in the study Hobbs et al. [17]. The following procedure coincided with the first evaluation method. Measurements were performed in duplicate and the results are expressed as mean values ± standard deviation.

#### 2.2.9. pH Leaching

pH leaching was determined as pH measurement of 2% solution. The water for measurement was degassed by 1 min of boiling. The pH of solution of CPE was measured using a surface pH microelectrode connected to a pH meter (pH 210, Hanna Instruments, Mauritius).

#### 2.2.10. Charge Density

To evaluate the charge density, 25 g of excipient was blended by a blender (Turbula T2C, Swiss) at 40 rpm in a glass container of 2 L volume. After 0, 5, 10, and 20 minutes of blending, excipient was transferred to Faraday pail (JCI 150, Chilworth Technology, Ltd.) connected to calibrated charge measurement unit (JCI 178, Chilworth Technology, Ltd.). Exact transferred mass of excipient was weighted afterwards. Charge density was obtained by dividing the net charge by the mass of excipient. Measurements were performed in duplicate and the results are expressed as mean values ± standard deviation.

#### 2.2.11. Scanning Electron Microscope (SEM)

The surface morphology of the CPE and compressed tablets was examined by SEM. The samples were attached to aluminium stubs with double side adhesive carbon tape and then gold coated with a sputter coater (JEOL NeoCoater MP-19020NCTR, Japan) and examined using a scanning electron microscope (JEOL JCM-6000, Japan). The signals of the samples were produced by backscattered electrons (BSE), at 15 kV voltage and different magnifications.

#### 2.2.12. DSC

DSC experiments were performed using testing machine (DSC 7 instrument, Perkin Elmer Instruments, USA). The heating rate and heat flow were calibrated at 10°C·min^−1^ using indium and zinc standard. The heat flow rate was set at 10°C·min^−1^ and inert nitrogen atmosphere (3.5 Bar) was employed. Approximately 5 mg of every sample was weighed in vented aluminium pan with crimp-on lid. All samples were analyzed over the temperature range 50–250°C except the ones containing guar gum due to early decomposition onset.

#### 2.2.13. Tablet Preparation

Material testing machine (Zwick/Roell T1-FRO 50, Zwick GmbH, Germany) with the compaction punches and die (Adamus HT, Machine Factor Group, Poland) was used to compress tablets with the cylindrical shape having a mass of 200 mg and the diameter of 7 mm. The used compression pressures were 78, 130, and 182 MPa and the compression rate was 0.5 mm·s^−1^. 55 tablets were compacted at each compression pressure from each material without any glidants or lubricants. Tablets were stored in a polyethylene bags for 24 hours before testing.

#### 2.2.14. Energy Evaluation of Compression Process

The force-displacement record was used to evaluate the energetic parameters. During the compression, the computer program testXpert V. 9.01 recorded the energy consumed for friction *E*_1_ (J), the energy consumed for plastic deformation *E*_2_ (J), and the elastic energy released during decompression *E*_3_ (J) [18]. The above-mentioned energies were used to calculate the plasticity (%) according to the following equation [19]:

(2) $$\begin{array}{c} PL=100*\frac{E_{2}}{E_{2}+E_{3}}. \end{array}$$

The results of measurements of 55 tablets are expressed as mean values ± standard deviation.

#### 2.2.15. Ejection Force

The ejection force was measured using material testing machine (Zwick/Roell T1-FRO 50, Zwick GmbH, Germany). After the compression, the lower punch was removed and the test was started. The ejection rate was 10 mm·min^−1^. The computer program testXpert V. 9.01 recorded the maximal ejection force for 10 tablets from each material. The results of 10 measurements are expressed as mean values ± standard deviation.

#### 2.2.16. Uniformity of Mass

20 randomly selected tablets from each sample were weighed individually on an analytical balance (HR-120, A&D Company, Japan). The results of 20 measurements are expressed as mean values ± standard deviation.

#### 2.2.17. Pycnometric Density of Tablets

The density of tablets was measured using the gas displacement technique with a helium pycnometer (AccuPyc II 1340, Micromeritics, USA). A completely dry and accurately weighed test cell was filled with the tablets and weighed again. The test cell containing the sample was sealed in the pycnometer and analysis commenced. Measurements were performed ten times and the results are expressed as mean values ± standard deviation.

#### 2.2.18. Tablet Height and Tensile Strength

The crushing force, diameter, and height of tablets were measured in 10 tablets using the hardness tester (8M, Dr. Schleuniger Pharmatron AG, Switzerland). The tensile strength of tablets was calculated using the following equation [20]:

(3) $$\begin{array}{c} TS=\frac{2*F}{\pi *d*h}, \end{array}$$

where TS (MPa) is the tensile strength, *F* (N) is the crushing force, *d* (mm) is the diameter of tablet, and *h* (mm) is the height of tablet. The results of 10 measurements are expressed as mean values ± standard deviation.

#### 2.2.19. Consolidation Behavior of Powders

The calculation of consolidation behavior of powders was based on the tablet volume (see (4)) which was related to the bulk volume of the same weight of powder (200 mg). For the calculation of percentage loss in volume (consolidation) (5) was used.

(4) $$\begin{array}{c} V=\pi \cdot r^{2}\cdot h, \end{array}$$

where *V* (cm^−3^) is the tablet volume, *r* (cm) is tablet radius, and *h* (cm) is the height of tablet.

(5) $$\begin{array}{c} consolidation=\frac{V_{b}-V_{t}}{V_{b}}\cdot 100, \end{array}$$

where *V*_*t*_ (cm^3^) is the volume of tablet and *V*_*b*_ (cm^3^) is the bulk volume of the powder.

#### 2.2.20. Friability

Approximately 6.5 g of dedusted tablets was weighed precisely using an analytical balance (HR-120, A&D Company, Japan) and placed into the plastic drum of a tablet friability tester (FT2, Sotax AG, Switzerland) and rotated for 4 minutes at 25 rpm in compliance with Ph. Eur. Thereafter, the dust was removed and tablets were reweighed. The loss of mass in percent for each tablets' sample was calculated.

#### 2.2.21. Disintegration Time

The disintegration test was performed on six tablets from each sample at 37.0 ± 2.0°C in distilled water using a disintegration test apparatus (ZT 301, Erweka GmbH, Germany). The tablets were considered completely disintegrated when no residue remained in the basket. Measurements were performed six times and the results are expressed as mean values ± standard deviation.

#### 2.2.22. Determination of Wetting Time and Water Absorption Ratio

Wetting time and water absorption ratio of tablets were determined in a Petri dish using a sponge (5 × 5 cm), impregnated with fifteen grams of water containing a water-soluble blue color for easier identification of complete wetting. The tested tablet was carefully placed on the surface of the impregnated sponge in the Petri dish at the laboratory temperature. The time required to reach the upper surface of the tablet (*T*_1_) and the time necessary for complete wetting of the tablet (wetting time, *T*_2_) by the color solution were noted. The weight of tablet in the dry state was noted as *m*_0_. The wetted tablet was carefully removed and reweighed using the analytical balance (*m*_1_). The water absorption ratio (WA) was calculated using the following equation:

(6) $$\begin{array}{c} WA=100*\frac{\left(m_{1}-m_{0}\right)}{m_{0}}. \end{array}$$

Measurements were performed five times and the results are expressed as mean values ± standard deviation.

## 3. Results and Discussion

Compositions of investigated CPE with different content of MCC and auxiliary excipients in binary mixtures are listed in Table 1. Avicel CE 15 contains medium part of MCC and guar gum, creating hydrocolloid in water; Avicel DG contains lower part MCC and water insoluble DCPA and finally Avicel HFE 102 contains higher part of MCC and highly soluble mannitol. Initially, all mixtures were characterized and tablets were made out of them without any additives. Both mixtures and tablets underwent a specific pharmacopoeial and physical evaluation set. Because all CPE are prepared by SpD, the composition should be the most important distinguishing factor of mixtures behavior. However, instead of correlating the effect of composition and processing technology on the measured properties, this article rather focuses on the comparison of characteristics relevant to use of these CPE for DC.

### 3.1. Powder Flow

Flow properties of mixtures were assessed by methods based on the mobility, for example, ability of particles to migrate, such as* flow through the orifice, angle of repose, *and* angle of slide*, and also by methods such as* CI* and* HR* that give information on behavior of powder density.

In* flow through the orifice* test, only Avicel HFE 102 provided measurable values as other samples did not pass through orifice at all (Table 2). Although ability to pass this test acceptably anticipates better results of mass uniformity of the prepared tablets, due to uniform filling of matrices, experimentally obtained data does not always confirm this assumption [21].

During the testing of the* angle of repose*, only Avicel HFE 102 showed measurable values (Table 2), evaluated as “good” by the pharmacopoeia. Powders, exhibiting such low values, are expected to be free of forming the cavities during the emptying of hopper and in the cavities of tablet matrices during compression. This should lead to better uniformity of mass and content [22].

Only* angle of slide *(Table 2) testing was able to evaluate all of the excipients, as it is used for difficultly flowing powders [23]. Flow abilities decrease in order of Avicel HFE 102 < Avicel CE 15 < Avicel DG. Preferred values of angle of slide are under 33°, because such powders do not tend to cling on the inside wall of hopper and do easily flow into the tablet cavities [24].

Indexes* HR* and* CI* (Table 2) are based on the ability of powder to decrease its apparent density and are evaluated by the comparison of* bulk* and* tapped density *(Table 2). Avicel HFE 102 and Avicel CE 15 showed “fair” values, while Avicel DG proved to be “passable” [25]. It is presumed that stable density of powder through compression results in smaller fluctuations in the mass of prepared tablets. But not even this is always true and therefore all evaluated CPE are considered to be acceptable [26].

Generally it is possible to say that both mobility and density aspects of flow properties increase in order of Avicel HFE 102 < Avicel CE 15 < Avicel DG.

### 3.2. Density and Porosity of Powders

Density of powder is mainly related to properties as dilution potential and the size of the tablet. Therefore, it is possible to diminish unsuitable properties of API by its more effective “diluting” due to compressing of greater quantity of the denser powder [27]. The* bulk* and* tapped densities* of all CPE are in the mid-range of powder classification [28]. Values of the* pycnometric density* (Table 2) of Avicel DG are almost doubled in comparison to the other types. This is probably caused by the presence of DCPA that is of higher density than guar gum and mannitol [29]. It is inherent that Avicel DG contains higher amount of air in its structure and therefore possesses the highest value of* porosity*. Otherwise porosity decreases in order of Avicel DG > Avicel HFE 102 > Avicel CE 15. High porosity of Avicel DG can be advantageously used for dry granulation, where double compression occurs in compaction and following tableting as trapped air allows repeated compression [30].

### 3.3. Particle Size and Specific Surface Area

The* size of particles*, or more precisely their distribution, can affect the uniformity of content in final tablets. This is due to a multiple facts. One of them is that major components in drug dosage tend to blend better when they are of comparable particle size and distribution [31]. On the contrary, minor components can benefit from relatively smaller particle sizes, by adhering onto the surface of majorly present and bigger particles [32].

**Figure 1:**
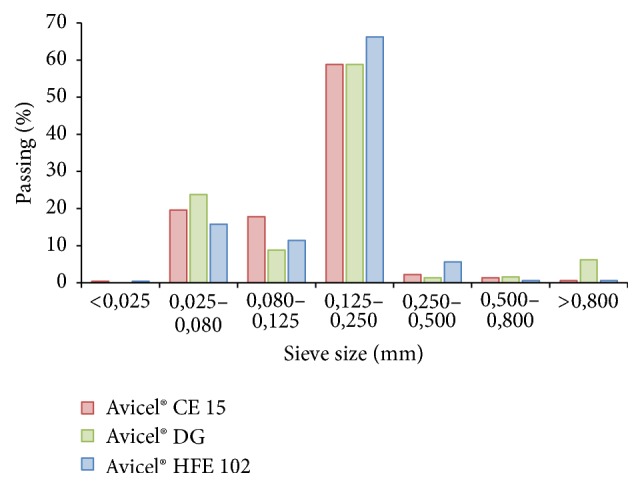
Particle size distribution by sieve analysis.

**Figure 2:**
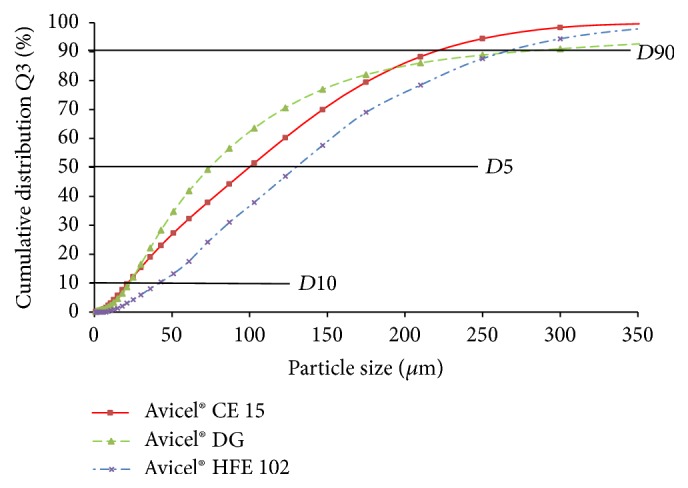
Particle size distribution by diffraction analysis.

Using the sieve analysis,* mean particle size* decreases in order of Avicel HFE 102 > Avicel DG > Avicel CE 15 (Figure 1); however, by comparing a *D*_50_ parameter obtained by laser diffraction, particle size decreases in the order of Avicel 102 > Avicel CE 15 > Avicel DG (Figure 2). Such discrepancy is usually assigned to differences in the principles of used methods. Distribution of the size fractions by sieve analysis (Figure 1) shows being uniform. When comparing the* specific surface area *(Table 2), which is inversely related to particle size, it decreases in order of Avicel HFE 102 > Avicel CE 15 > Avicel DG, confirming the laser diffraction to be more precise [33].

### 3.4. Moisture Content, Hygroscopicity, Solubility, and pH Leaching

**Figure 3:**
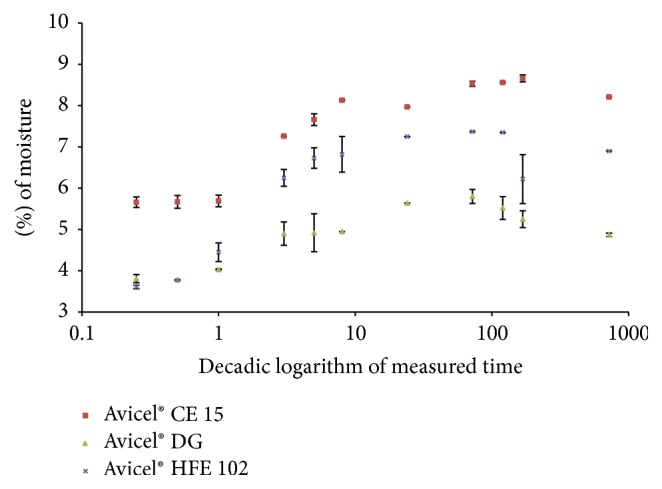
Hygroscopicity of CPE.

*Moisture content* of all CPE lies comparably in the range between 3 and 5%, increasing in order of Avicel HFE 102 < Avicel DG < Avicel CE 15 (Table 2). More pronounced is the difference in* hygroscopicity* (Figure 3), which increases in order Avicel DG < Avicel HFE 102 < Avicel CE 15. This is probably due to the fact that DCPA does not rehydrate at given conditions [34] and that mannitol is not hygroscopic compared to guar gum [34, 35]. In conclusion Avicel CE 15 is less suitable for the formulation with hydrolyzing substances. On the contrary Avicel DG seems to be the best choice for formulations with high content of humidity sensitive substances, especially when combined with dry granulation.

Solubility-wise, amount of* undissolved fraction after 3 minutes* (Table 2) in conditions simulating oral cavity is almost identical in case of all Avicel types. As this amount does not exceed 10%, it is obvious that these CPE are not suitable for the formulation of orodispersible tablets. However, total* undissolved fraction after 24 hours* increases in order of Avicel CE 15 < Avicel DG < Avicel HFE 102 (Table 2).

Neutral pH of Avicel HFE 102 and Avicel DG infusions is to be linked to the content of neutral DCPA and mannitol. On the other hand, Avicel CE 15 is weakly acidic, probably due to a presence of guar gum [36]. This fact may limit the use of Avicel CE 15, when substances hydrolyzing in slightly acidic conditions are to be used in drug dosage formulation.

### 3.5. Charge Density

**Figure 4:**
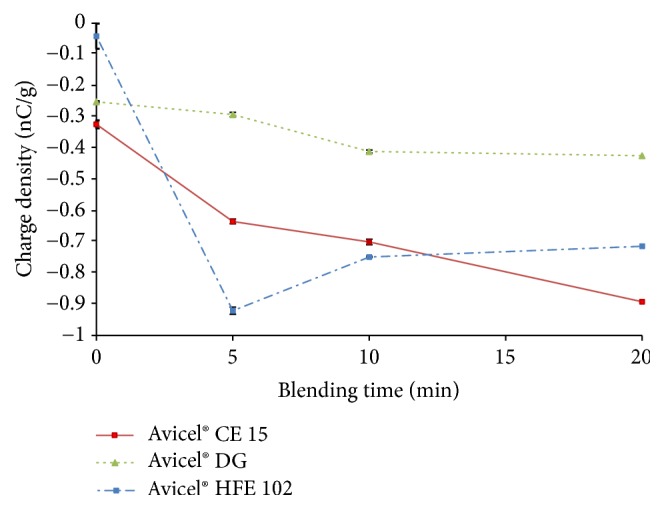
Charge density of CPE throughout blending.

Throughout manufacturing and handling interactions occur upon contact or friction among particles of excipients and APIs or between the particles and surfaces in contact. These interactions are able to induce electrostatic charge in mixtures, affecting formulation, manufacturing process, and packing behavior, as well as influencing mass and content uniformity of products. For these reasons* charge density* of excipients was examined. All excipients exhibited increasing negative charge density throughout blending as displayed Figure 4. The presence of uncontrolled electrostatic charges may have an adverse effect on powder blend uniformity. In contrast, blending of oppositely charged excipient material and API material can lead to a better blend uniformity [37].

### 3.6. Scanning Electron Microscope (SEM)

**Figure 5:**
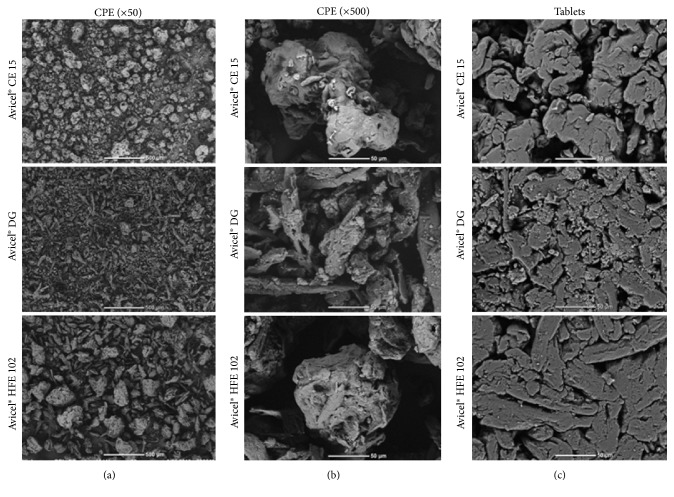
SEM pictures of CPE (a and b) and tablets (c).

Looking at 50x magnification in Figure 5, it can be seen that Avicel DG shows nearly no spherical particles, despite the fact that all of these excipients are prepared by SpD, which is prone to produce spherical structures. The low spherical profile is caused probably by use of high amount of DCPA, which is represented by small white particles, which are not able to pack fibrous structures of MCC on themselves and thus create spheres. This poor spherical profile of Avicel DG is an indicator of the poorest flow properties among the analyzed excipients. Somewhat more spherical particles exhibit Avicel CE 15, which also corresponds to its better flow properties. Avicel HFE 102 has the most relatively regular particles, though it is not even possible to talk about spheres here. Therefore, its flow properties are significantly higher than the other two types. The direct relationship between particle sphericity and flow properties of SpD particles is known from the literature [38].

500x magnification (Figure 5) shows particles of CPE in detail. There are visible DCPA particles present on the particles of MCC in Avicel DG. Particles of Avicel HFE 102 look like typical particles made by SpD: conglomerated particles of MCC and mannitol can be easily differentiated in primary particles. As for Avicel CE 15, the primary particles exhibit small cavities in them and look like rolled fibers of MCC glued together with guar gum. Guar gum is possibly the main reason of almost no presence of free MCC fibers because it is used as a binder [39].

Comparing surfaces of tablets, it is clearly seen that excipients with high content of cellulose are prone to produce moldings with regular and smooth surfaces [40]. The size of pores is again determined by content of MCC [41], whereas smallest pores are present for Avicel HFE 102 with the highest amount of MCC. According to this theory, Avicel CE 15 should have slightly bigger pores than Avicel HFE 102, smaller than Avicel DG. This, however, is not observed in the presented pictures. But since Avicel DG contains small particles of DCPA tending to fit between fibers of MCC, it can skew this assumption.

### 3.7. DSC

**Figure 6:**
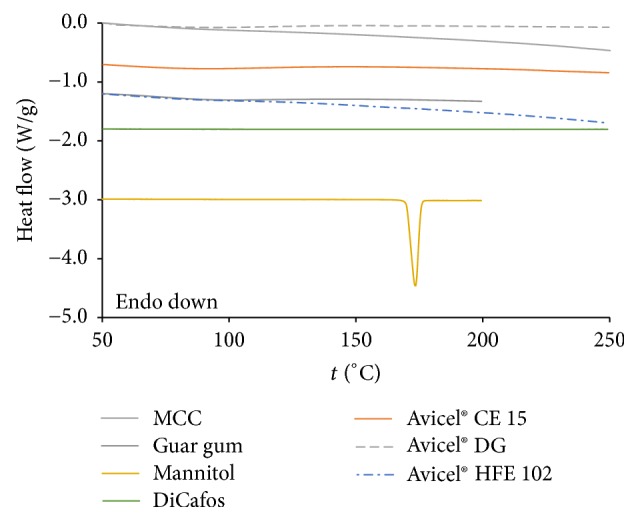
DSC analysis of components.

*DSC analysis* was performed to assess, namely, the crystalline state of individuals in mixture, to distinguish the presence of crystalline water or residual moisture, and to eventually identify ongoing interactions between individuals. All obtained DSC curves are displayed in Figure 6 and they are stepped by 0.6 W/g. The DSC curves of Avicel DG and Avicel CE 15 show no characteristic peaks except for broad endothermic peak in 50–120°C region associated with moisture desorption from MCC. In case of Avicel CE 15 it is probably combined with guar gum endothermic peak which is broad also. DSC curve of Avicel HFE 102 lacks typical melting endotherm of mannitol (onset temperature 169.9°C). Taking in account the cospray processing of this multiexcipient, we assumed that mannitol is present in amorphous state. These profiles may be useful for tablet manufacturers to compare potential drug interactions with individual CPE.

### 3.8. Energetic Parameters of Compression Process

Values of energetic parameters *E*_1–3_ are shown in Table 3. All measured energetic parameters increased with increasing compression pressure. The highest values in all samples were found for* energy E*_1_ (energy consumed for friction). This energy may be associated with rearrangement, size, and shape of particles and should be as small as possible in favour of energy *E*_2_ (energy consumed for plastic deformation) [42]. The lowest values were measured for Avicel CE 15 and in other samples values increase in order Avicel DG < Avicel HFE 102. As can be seen in Figure 3, particles of Avicel CE 15 are of almost spherical shape with only small irregularities on their surface. Particles fit better together and fill the empty spaces, which can lead to lower energy consumption during rearrangement. On the contrary, the surface of Avicel HFE 102 particles is more regular; however, they are also bigger than those of other used excipients (Table 2). Particles may hook during precompression phase and that results in higher value of energy *E*_1_. Particles of Avicel DG have the most irregular shape; however, they are also the smallest ones of used excipients. As a result, the hooking occurs in lesser extent and the energy *E*_1_ is smaller than in the case of Avicel HFE 102.

*Energy E*_2_ represents the amount of energy used for the plastic deformation of particles and friction between the particles and die wall [18]. The highest values for Avicel CE 15 can be caused by a combination of two well plastically deforming materials. MCC especially subjects to plastic deformation [7] as well as guar gum. The important role has also the ability of these materials to create hydrogen bonds [43] or mechanical interlocking among particles during compression [44, 45]. Nevertheless, according to Inghelbrecht and Remon [11], granules prepared from Avicel CE 15 have very low hardness and addition of guar gum did not improve its value. It also corresponds to low tensile strength of tablets made of Avicel CE 15 (Figure 8). Lower values for *E*_2_ parameter were measured for Avicel DG and Avicel HFE 102. However, there is no significant difference between these two CPE. They both contain MCC and mannitol or DCPA. DCPA is considered as a brittle material with high fragmentation of particles during compression [46]. The combination of plastic and brittle material probably decreases the amount of hydrogen bonds due to their replacement by the van der Waals forces. Higher amount of smaller particles and hence greater surface area are formed during the roll compaction due to the fragmentation of DCPA. Subsequently newly created surfaces are available for bonds formation during compression. Avicel DG is thus suitable for dry granulation process. Mannitol is considered as a ductile material [47]; however, particles of Avicel HFE 102 break under compression and the deformation is more similar to Avicel DG (Figure 4).

*Parameter E*_3_ expresses the elastic energy of the compressed material. It is the energy released after compression [48, 49]. Only small differences in the values of *E*_3_ parameter can be observed among used excipients. The highest values were measured for Avicel CE 15. This can be caused by good viscoelastic properties of MCC and guar gum. Avicel DG and Avicel HFE 102 have almost the same values of this parameter. Lower *E*_3_ than Avicel CE 15 can be given by the presence of mannitol or DCPA, which have poor ability to have elastic deformation [50, 51].

### 3.9. Plasticity

*Plasticity* (Pl) is calculated according to Stamm and Mathis [19] and describes the relation between reversible and irreversible deformation. The amount of energy consumed for plastic deformation depends mainly on the properties of compressed material. A high Pl value reflects the high amount of energies used for irreversible deformation of the compressed material.

The values of Pl at compression pressure of 78 MPa decrease in order Avicel DG > Avicel HFE 102 > Avicel CE 15 (Table 3). However, higher values were found for Avicel CE 15 and Avicel HFE 102 when compression pressures of 130 and 182 MPa were employed. Avicel CE 15 has high values of energy used for the plastic deformation *E*_2_ and hence higher plasticity Pl. Lower plasticity in Avicel DG at higher compression pressures can be also explained by lower *E*_2_ values.

### 3.10. Ejection Force

*Ejection force* is the force developed by upper punch required to eject the tablet from the die. It can be affected by several factors, for example, friction between particles and die wall or ejection speed [52]. For all used excipients, the ejection force increases with increasing compression pressure from 78 to 130 MPa (Table 3). Moreover, in the case of Avicel CE 15 and Avicel HFE 102 tablets ejection force also increases with increasing compression pressure from 130 to 182 MPa. For Avicel CE 15 tablets the values of ejection force are the lowest ones of used excipients. This is related to the good self-lubricating properties of Avicel CE 15. Ejection forces of Avicel DG increase with increasing compression pressures and are the highest ones of measured results, except the value measured at 182 MPa, where the significant decrease of ejection force occurs. For Avicel HFE 102 the value increases with increasing compression pressure and is similar to Avicel DG. This is consistent with the results of Westerhuis et al. [53]. In their study it was found that in the granules containing mannitol and 90% of MCC the ejection force increases with increasing compression force. Higher values of ejection force in the case of Avicel DG and Avicel HFE 102 can be caused by the combination of excipients. MCC as the viscoelastic filler shows adhesion to the die wall, mannitol, and DCPA as plastic/brittle fillers have tendency to adhere to punch faces, which can cause sticking and increase in ejection force [54]. Mannitol itself also exhibits high frictional force during tablet ejection [55, 56]. The ejection force can be also related to capping problems as was reported by Van Der Voort Maarschalk et al. [57]. The capping probability is thus higher for these two materials in comparison to Avicel CE 15.

### 3.11. Uniformity of Mass

The evaluation of weight variation implied that none of the prepared tablets deviated from the average value by more than 7.5% (Table 4), which is the limit given by Ph. Eur. [58]. The evaluation of the* uniformity of mass *was done only for the control of similar compaction condition, because each tablet was prepared separately using the Zwick/Roell T1-FRO 50 machine without the automatic filling of the die from the hopper. Therefore, deterioration of the mass uniformity of tablets prepared from Avicel CE 15 and Avicel DG without the use of lubricants (e.g., magnesium stearate) can be expected considering their inferior flow properties (Table 2).

### 3.12. Pycnometric Density of Tablets

The highest value of* pycnometric density* was observed in tablets containing Avicel DG (Table 4), which implied also the highest pycnometric density in the form of powder as was discussed above. Tablets containing Avicel CE 15 and Avicel HFE 102 have similar pycnometric density. Moreover, it was observed in samples of Avicel DG and Avicel HFE 102 that value of pycnometric density decreases with increasing compression pressure. This observation can be explained with decreasing porosity of tablets prepared at higher pressures. Surprisingly, values of pycnometric density of tablets containing Avicel CE 15 increase with increasing compression pressure. Similar results for tablets containing MCC were observed by Sun [59]. In this study Avicel PH 102 was compressed in different compaction pressures. It was observed that the highest value of pycnometric density was achieved at 100 MPa and then with increasing pressure densities stay constant [59].

### 3.13. Tablet Height and Consolidation Behavior of Powders

The evaluation of* tablet height* revealed that with increasing compression pressure the height of tablets decreases. This phenomenon was observed in all materials as could be seen in Table 4. The height of tablets was in order of Avicel DG < Avicel HFE 102 < Avicel CE 15. This observation can be connected to the different elastic recovery of each material. The highest values of elastic energy (*E*_3_) of Avicel CE 15 indicate the greatest elastic recovery and hence the highest tablets. On the other hand, Avicel DG and Avicel HFE 102 indicate lower values of *E*_3_ and, therefore, lower tablets were obtained.

**Figure 7:**
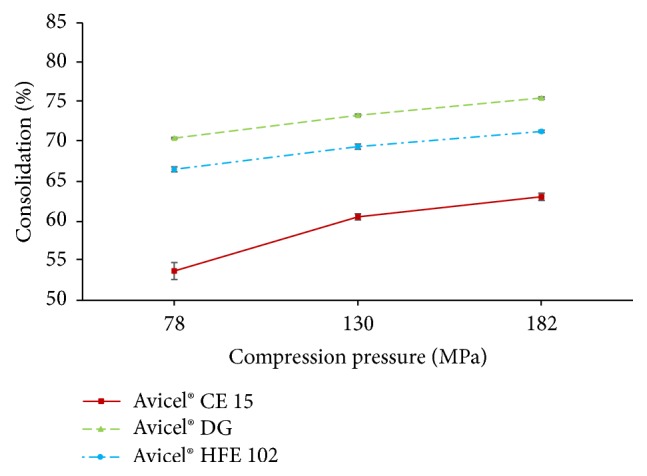
Consolidation behavior.

The highest percentage loss of volume during compression was observed in Avicel DG tablets (Figure 7). This Avicel type contains higher amount of air in its structure and therefore implies higher loss of volume during compression. The degree of consolidation was in order Avicel CE 15 < Avicel HFE 102 < Avicel DG. However, the highest decrease in tablet height between tablets prepared using compression pressure of 78 and 182 was observed in Avicel CE 15 (19.13%), while the lowest one was observed in Avicel HFE 102 (12.4%). This observation may be explained by the presence of guar gum in the Avicel CE15 composition, which is more elastic in comparison to brittle material in composition of Avicel DG and Avicel HFE 102 (DCPA and mannitol).

### 3.14. Tensile Strength

Tablet hardness serves both as a criterion to guide product development and as a quality-control specification. According to the scientific literature [60, 61], the optimal value of* tensile strength* of tablet should lie within the range of 0.56 and 1.12 MPa. However, each material has its unique characteristics and therefore even tablets having tensile strength lower than 0.56 MPa or higher than 1.12 MPa can have appropriate properties for manufacturing, handling, and application to patients as was proved in several studies [62–64].

**Figure 8:**
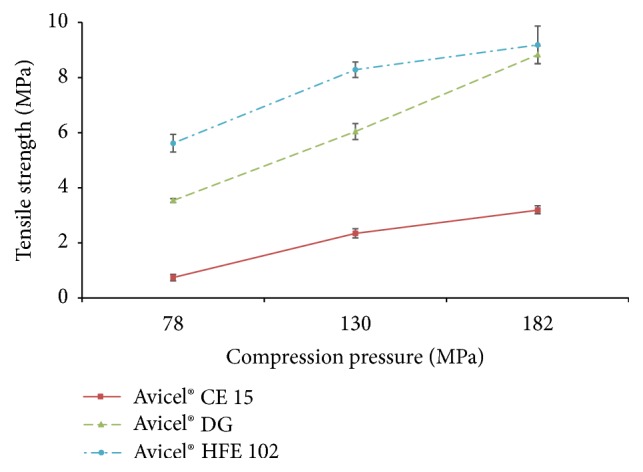
Tensile strength of tablets.

Tensile strength of all tablets increases with increasing compression pressure as can be seen in Figure 8. Only tablets containing Avicel CE 15 prepared using compression pressure of 78 MPa have the value of tensile strength in the recommended range (0.56–1.12 MPa). However, these tablets also have a high value of friability (2.56%) and therefore do not meet the pharmacopoeial requirements. The rest of the samples show the value of tensile strength above the recommended range that can predict good mechanical endurance of tablets even when excipients with negative effect on tablet hardness (e.g., lubricants [65]) are used.

The lowest tensile strength was observed in tablets containing Avicel CE 15. This finding is consistent with results obtained in the study of Inghelbrecht and Remon [11]. The addition of guar gum to MCC lowered granule quality and the tablet hardness was the lowest in comparison to other tested types of Avicel.

Tablets containing Avicel DG and Avicel HFE 102 imply higher values of tensile strength (Figure 8) in comparison to Avicel CE 15. This observation can be explained by the fragmentation of particles. New surfaces are created during the compression process of Avicel DG and Avicel HFE 102 and thus more bonds among particles can be employed to strengthen the structure of tablet. Moreover, particles of Avicel HFE 102 are fragmented into greater amount of smaller pieces (Figure 4); hence, its tensile strength is higher in comparison to Avicel DG.

### 3.15. Friability

The measured values of* friability* are shown in Table 4. Evaluation of tablets prepared using Avicel CE 15 revealed that with increasing compression pressure the value of friability decreases. However, tablets prepared using lowest compression pressure showed friability of 2.56% and therefore do not meet requirements for friability testing. The other two excipients exhibit lower values of friability. The slightly lower values were observed in tablets containing Avicel DG when higher compression pressures were employed. Compaction of Avicel HFE 102 leads to tablets with the lowest friability. Similar results were observed by Daraghmeh et al. [66]. In their study, tablets containing Avicel HFE 102 also implied low values of friability (lower than 0.5%). Moreover, it was confirmed that with increasing values of tablet hardness (crushing force) the friability decreases [66]. Also, tablets containing different coprocessed mixtures with varying amounts of MCC and mannitol prepared by Jacob et al. exhibited very low friability (less than 0.5%) [67].

### 3.16. Disintegration Time

The* disintegration times* of all prepared tablets are shown in Table 4. The evaluation of disintegration time of tablets revealed that increasing of compression pressure leads to prolongation of disintegration time. This phenomenon may be explained by the presence of MCC, which has a very high intraparticle porosity with about 90–95% of the surface area being internal. This high porosity promotes the process of swelling and disintegration of MCC tablets. Higher tableting pressure leads to lower porosity of tablets and, therefore, decreases water penetration into tablets and hence increases disintegration time [68]. These results also correspond to increasing values of tablets tensile strength (Figure 8), which very often leads to slower disintegration [69]. Prolonging of the disintegration time can be expected with increased tablet hardness, due to the stronger intraparticular bonds [70].

The disintegration rate was in order of Avicel HFE 102 > Avicel CE 15 > Avicel DG. The only exceptions were tablets containing Avicel DG prepared using the pressure of 182 MPa, which do not disintegrate within 60 minutes and, therefore, do not meet the compendial requirements for disintegration of uncoated tablets. On the other hand, tablets compressed at lower pressures imply very fast disintegration within 2 minutes and may be described as fast disintegrating tablets. This could be explained by the presence of DCPA, which allows rapid and complete penetration of the liquid into the tablets due to its hydrophilic nature and the high porosity of the tablets. Despite the fast and complete water penetration, tablets containing only DCPA do not disintegrate because the excipient is relatively insoluble in water [71]. Nevertheless, water penetration enhances the disintegration properties of MCC in tablets prepared at lower compression pressures. However, tablets compressed at 182 MPa have lower porosity and slower penetration of water and hence longer disintegration of tablets was observed (Table 4). In the case of Avicel HFE 102 faster disintegration can be expected when higher amounts of mannitol are used as was described by Jacob et al. [67]. In their study it was observed that higher amount of mannitol in the coprocessed mixture of mannitol and MCC decreases the disintegration time. This phenomenon can be explained by high hydrophilicity of the mannitol and also by its high solubility in water [29].

### 3.17. Water Absorption Ratio and Wetting Times

**Figure 9:**
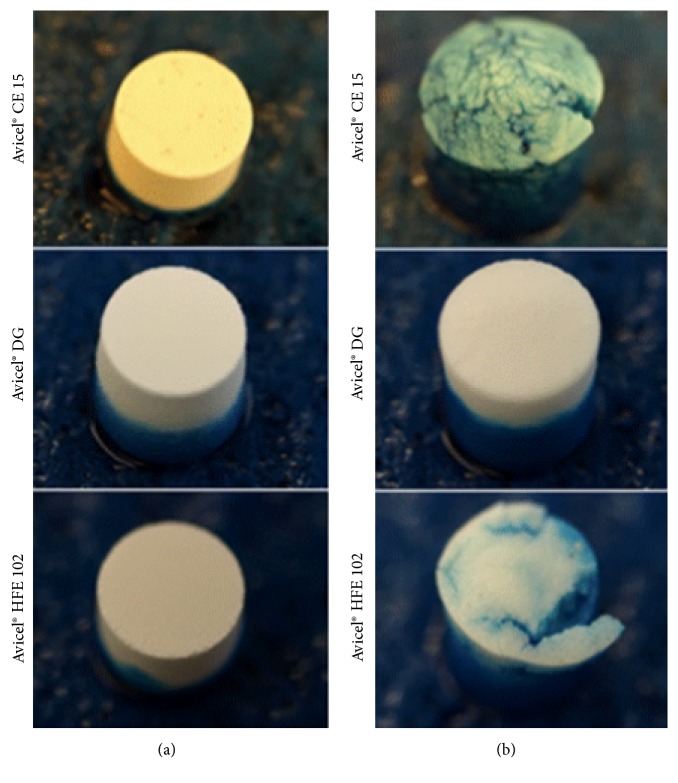
Tablet prepared using compression pressure of 78 MPa before (a) and after (b) complete wetting.

It is already known that* wetting time* is closely related to the inner structure of the tablets and to the hydrophilicity of the excipients used. It has also been observed that wetting time often increases with an increase in compression pressure, smaller pore sizes, and decrease in porosity [72]. The results, obtained from the evaluation of wetting times and* water absorption ratio*, are presented in Table 4. The differences in samples prepared using compression pressure of 78 MPa before and after complete wetting can be seen in Figure 9.

All tablets containing Avicel CE 15 show longest wetting times and highest values of WA in comparison with other formulations. The longer wetting times were observed with increasing compression pressure, while the values of water absorption ratio were similar in all samples. This finding can be explained by the fast water wicking rate of MCC [73] and swelling of guar gum as is also visible in wetted tablets (Figure 9). The swelling action of guar gum is controlled by the rate of water uptake into the matrices [74], which is partially enhanced by the presence of MCC. However, porosity of tablets decreases with increasing compression pressure, which slows down the penetration of water and hence wetting and swelling of guar gum particles. The swelling properties of guar gum also affects the value of WA, which is the greatest one of all measured tablets (more than 350%).

The evaluation of wetting time revealed that tablets containing Avicel DG exhibit very fast wetting times. The wetting times are also prolonged with increasing compression pressure due to decreasing porosity of tablets. This observation can be explained by the presence of dicalcium phosphate, which allows rapid and complete penetration of the liquid into the tablet due to its hydrophilic nature and the high porosity of the tablets [71] as has been mentioned before [75].

The same dependence of the compression pressure on the wetting time was observed in Avicel HFE 102 tablets. The results also correspond to the prolonged disintegration time and increasing hardness of these tablets. Kalia et al. [76] compared the properties of tablets containing dicalcium phosphate, mannitol, or MCC as a diluent. The results showed that tablets containing mannitol had faster wetting time in comparison to tablets with dicalcium phosphate and MCC (54.3 ± 2.51 seconds). The probable reason for faster wetting of mannitol tablets might be its water-soluble nature and its presence in the form of large granular particle [34]. The water absorption ratio of Avicel HFE 102 tablets decreases with increasing compression pressure (Table 4). This reduction of water absorption ratio can be caused by the longer wetting time. When the tablet was placed on the sponge for the longer time, dissolving of the mannitol occurred. Also Kalia et al. [76] observed that mannitol disintegrates instantaneously on contact with saliva. The tendency of tablets to disintegrate during the evaluation of wetting properties can be seen in Figure 9.

## 4. Conclusion

Three widely used types of Avicel, based on the spray dried coprocessed mixtures, and the compressed tablets without the addition of lubricant were thoroughly examined for the physical characteristics. Avicel CE 15 has mid-range flow properties and has mid-sized and middle asymmetrical particles, high hygroscopicity, growing negative charge, best lubricity, lowest tensile strength, and mid-long disintegration times. It also has a superior organoleptic performance in chewable tablets. In comparison with other binders, guar gum provides good sensory experience and perceived taste and is not suitable for pH sensitive substances. Avicel DG possesses the worst flow properties, smallest and most asymmetrical particles, low hygroscopicity, stable charge, intermediate lubricity, and tensile strength and exhibits fast disintegration of tablets. It is suitable for dry granulation process of water sensitive substances. Avicel HFE 102 has the best flow properties, large and most symmetrical particles, and middle hygroscopicity and its charge fluctuates throughout blending. It also exhibits the worst lubricity, the highest tensile strength, and slow disintegration of tablets. It offers improvements in flow properties in combination with poorly flowing substances. All types are dedicated for per oral, nonorodispersible administration.

## Figures and Tables

**Table 1:** Composition of CPE.

Avicel CE 15	85% MCC, 15% guar gum
Avicel DG	75% MCC, 25% DCPA
Avicel HFE 102	90% MCC, 10% mannitol

**Table 2:** Physical characteristics of CPE.

Measured value	Avicel CE 15	Avicel DG	Avicel HFE 102
Fw [g·s^−1^]	NA	NA	11.2 ± 0.16
*θ*r [°]	NA	NA	30.67 ± 0.72
*θ*s [°]	39.00 ± 0.81	40.67 ± 0.47	37.33 ± 0.47
HR	1.24	1.33	1.24
CI	19.46	24.98	19.19
DB [g·mL^−1^]	0.50 ± 0.17	0.38 ± 0.26	0.41 ± 0.25
DT [g·mL^−1^]	0.62 ± 0.28	0.50 ± 0.37	0.51 ± 0.31
DP [g·mL^−1^]	1.40 ± 0.11	2.86 ± 0.12	1.58 ± 0.41
*P* [%]	64.3	86.7	74.1
MPS [*µ*m]	111.8	123.0	145.0
*D*_10_ [*µ*m]	21.55	22.55	41.93
*D*_50_ [*µ*m]	99.73	74.38	132.16
*D*_90_ [*µ*m]	221.06	276.8	267.37
SSA [m^2^·g^−1^]	0.5	1.2	0.6
*M* [%]	4.66 ± 0.13	3.35 ± 0.10	3.28 ± 0.04
UF_3_ [%]	98 ± 0.01	92 ± 0.00	95 ± 0.02
UF_24_ [%]	26 ± 0.08	65 ± 0.05	89 ± 0.43
pH	5.52	7.19	6.99

Fw: flow through the orifice; *θ*r: angle of repose (tg *α*); *θ*s: angle of slide; HR: Hausner ratio; CI: compressibility index; DB: bulk density; DT: tapped density; DP: pycnometric density; *P*: porosity; MPS: mean particle size; *D*_10_, *D*_50_, and *D*_90_: diameters at which 10, 50, and 90% of the sample's mass are comprised of particles with a diameter less than this value; SSA: specific surface area; *M*: moisture content; UF_3_: undissolved fraction after 3 min; UF_24_: undissolved fraction after 24 hours; pH (2% solution); N/A: not applicable.

**Table 3:** Energetic parameters of compression, plasticity, and ejection force.

Measured value	CP [MPa]	Avicel CE 15	Avicel DG	Avicel HFE 102
*E*_1_ [J]	78	3.15 ± 0.24	7.42 ± 0.15	8.03 ± 0.23
	130	7.99 ± 0.47	13.60 ± 0.24	15.05 ± 0.47
	182	13.29 ± 0.33	22.09 ± 0.67	22.60 ± 0.49

*E*_2_ [J]	78	3.72 ± 0.05	3.50 ± 0.03	3.49 ± 0.07
	130	5.50 ± 0.09	4.91 ± 0.17	5.07 ± 0.15
	182	6.76 ± 0.14	5.62 ± 0.18	5.77 ± 0.25

*E*_3_ [J]	78	0.30 ± 0.01	0.25 ± 0.01	0.26 ± 0.01
	130	0.63 ± 0.03	0.60 ± 0.01	0.58 ± 0.01
	182	1.28 ± 0.03	1.17 ± 0.03	1.15 ± 0.04

Pl [%]	78	92.54 ± 0.24	93.25 ± 0.08	93.06 ± 0.27
	130	89.67 ± 0.44	89.18 ± 0.54	89.68 ± 0.42
	182	84.04 ± 0.37	82.82 ± 0.73	83.42 ± 1.03

EF [N]	78	44.31 ± 1.59	234.36 ± 16.97	232.33 ± 9.42
	130	54.93 ± 2.35	276.46 ± 9.23	258.19 ± 12.62
	182	76.76 ± 4.04	166.80 ± 46.71	269.95 ± 62.89

CP: compression pressure; EF: ejection force.

**Table 4:** Properties of tablets.

Measured value	CP [MPa]	Avicel CE 15	Avicel DG	Avicel HFE 102
UM [mg]	78	198.15 ± 1.55	200.46 ± 0.50	200.97 ± 0.31
	130	200.30 ± 0.87	200.55 ± 0.40	200.84 ± 0.30
	182	199.78 ± 0.42	199.53 ± 0.54	199.18 ± 0.35

PD [g/cm^3^]	78	1.5284 ± 0.0004	1.7452 ± 0.0004	1.5486 ± 0.0002
	130	1.5360 ± 0.0003	1.7425 ± 0.0002	1.5446 ± 0.0001
	182	1.5420 ± 0.0002	1.7436 ± 0.0002	1.5504 ± 0.0002

He [mm]	78	4.81 ± 0.10	4.00 ± 0.01	4.18 ± 0.04
	130	4.16 ± 0.04	3.61 ± 0.02	3.84 ± 0.03
	182	3.89 ± 0.03	3.38 ± 0.01	3.66 ± 0.02

Fr [%]	78	2.56	0.10	0.01
	130	0.58	0.03	0.07
	182	0.28	0.03	0.04

Di [min]	78	2.15 ± 0.08	0.28 ± 0.00	4.03 ± 1.07
	130	7.27 ± 2.33	1.63 ± 0.68	15.30 ± 2.18
	182	11.12 ± 0.28	>60.0	21.42 ± 0.90

*T*_1_ [min]	78	8.27 ± 3.63	0.20 ± 0.03	0.70 ± 0.03
	130	38.52 ± 5.63	0.18 ± 0.00	7.46 ± 2.22
	182	66.73 ± 7.63	0.88 ± 0.07	17.75 ± 3.73

*T*_2_ [min]	78	10.25 ± 5.43	0.37 ± 0.08	0.96 ± 0.05
	130	55.62 ± 7.83	0.22 ± 0.02	10.57 ± 1.37
	182	94.48 ± 16.60	1.23 ± 0.32	28.27 ± 6.77

WA [%]	78	355.18 ± 16.43	90.74 ± 14.96	114.96 ± 9.43
	130	377.66 ± 6.71	112.30 ± 7.83	96.30 ± 27.22
	182	370.99 ± 7.93	64.70 ± 2.13	94.62 ± 18.27

CP: compression pressure; UM: uniformity of mass; PD: pycnometric density; He: height; Fr: friability; Di: disintegration; *T*_1_, *T*_2_: wetting time; WA: water absorption ratio.
